# Supplementary material for: Seminoma and Embryonal Carcinoma Footprints Identified by Analysis of Integrated Genome-Wide Epigenetic and Expression Profiles of Germ Cell Cancer Cell Lines
Source: PLoS One. 2014 Jun 2;9(6):e98330. doi: 10.1371/journal.pone.0098330 (PMC4041891; doi:10.1371/journal.pone.0098330)
Supplement: File S1 — ZIP file containing DMRforPairs output for significant regions. Please start from the html files. (ZIP) [file pone.0098330.s008.zip › figures/22595.pdf]

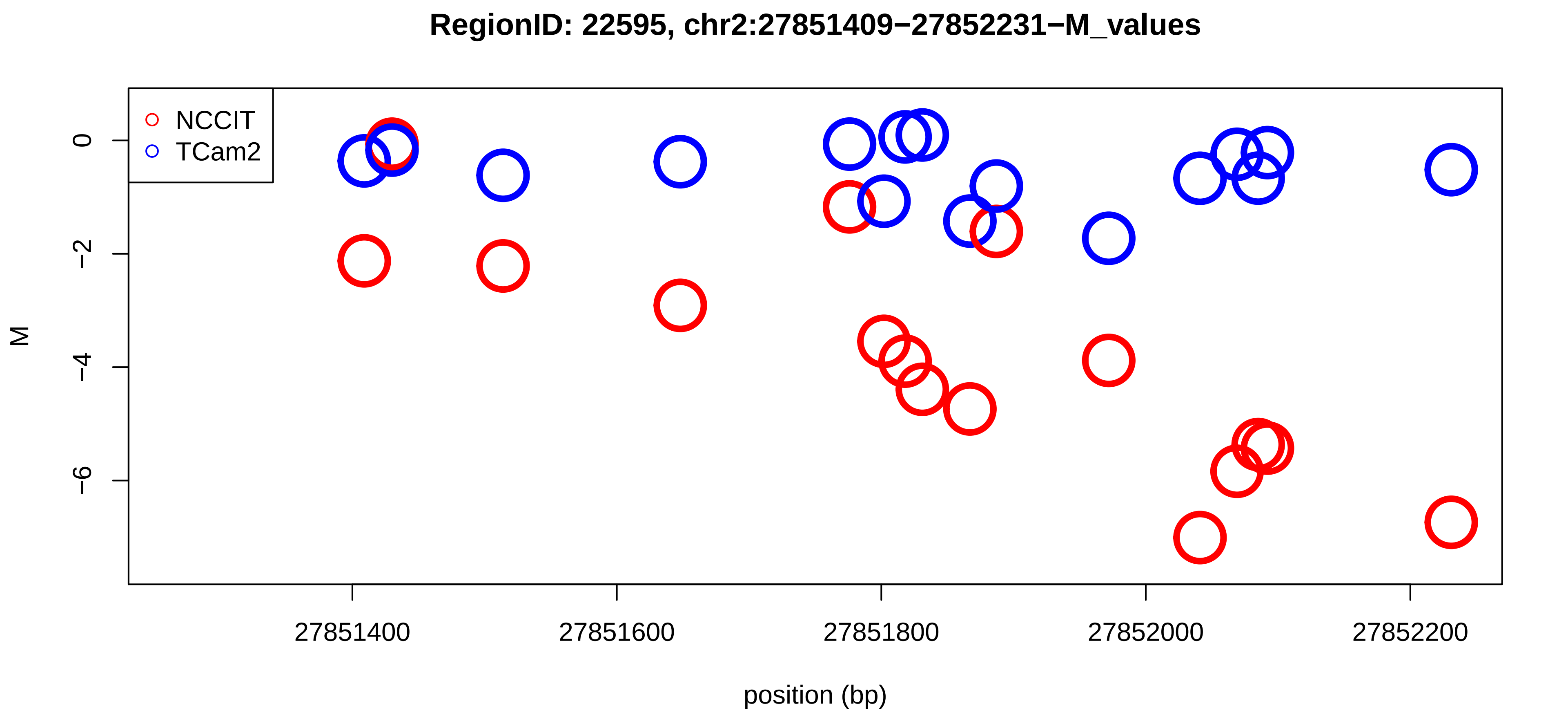

RegionID: 22595, chr2:27851409−27852231−M_values
NCCIT
TCam2
M
0
−2
−4
−6
27851400
27851600
27851800
27852000
27852200
position (bp)

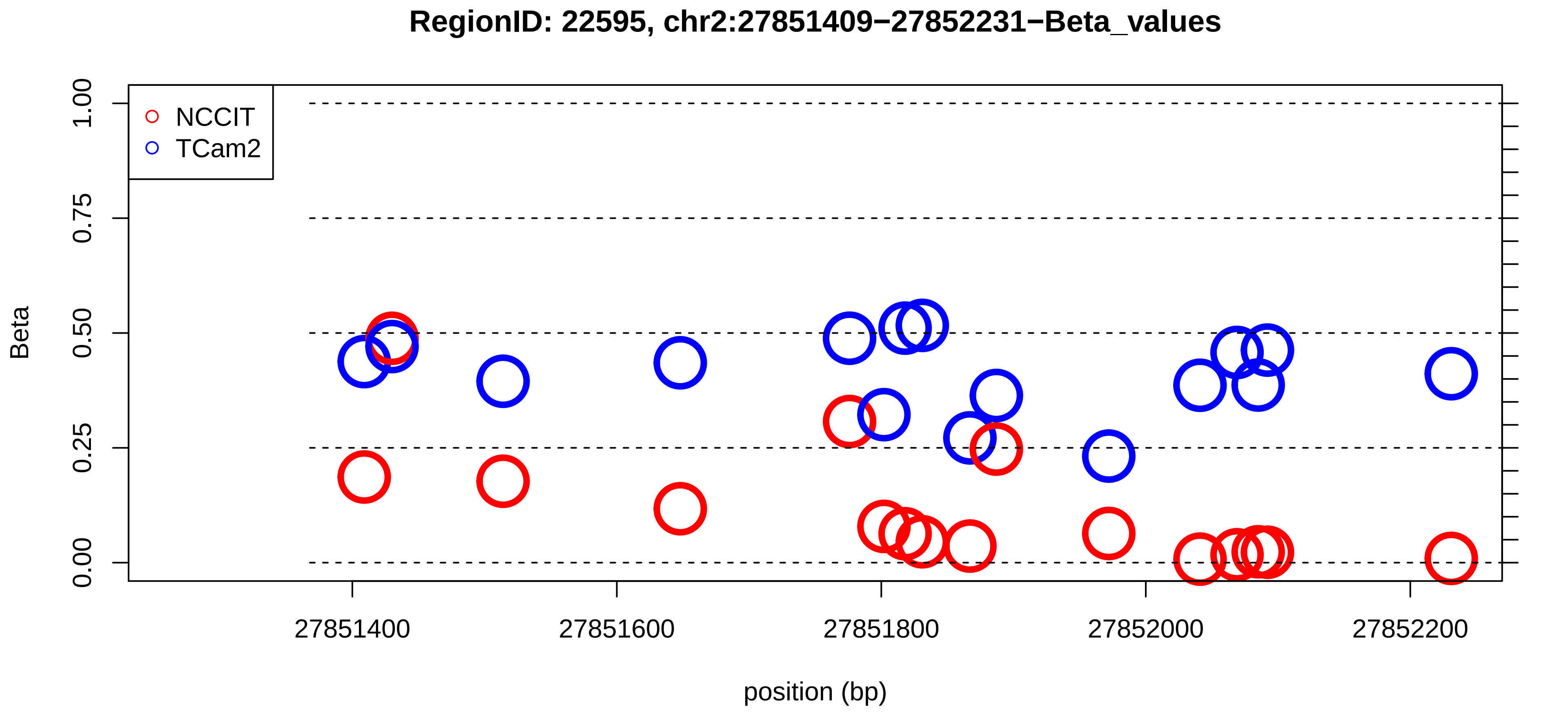

RegionID: 22595, chr2:27851409−27852231−Beta_values
NCCIT
TCam2
Beta
0.00
0.25
0.50
0.75
1.00
27851400
27851600
27851800
27852000
27852200
position (bp)

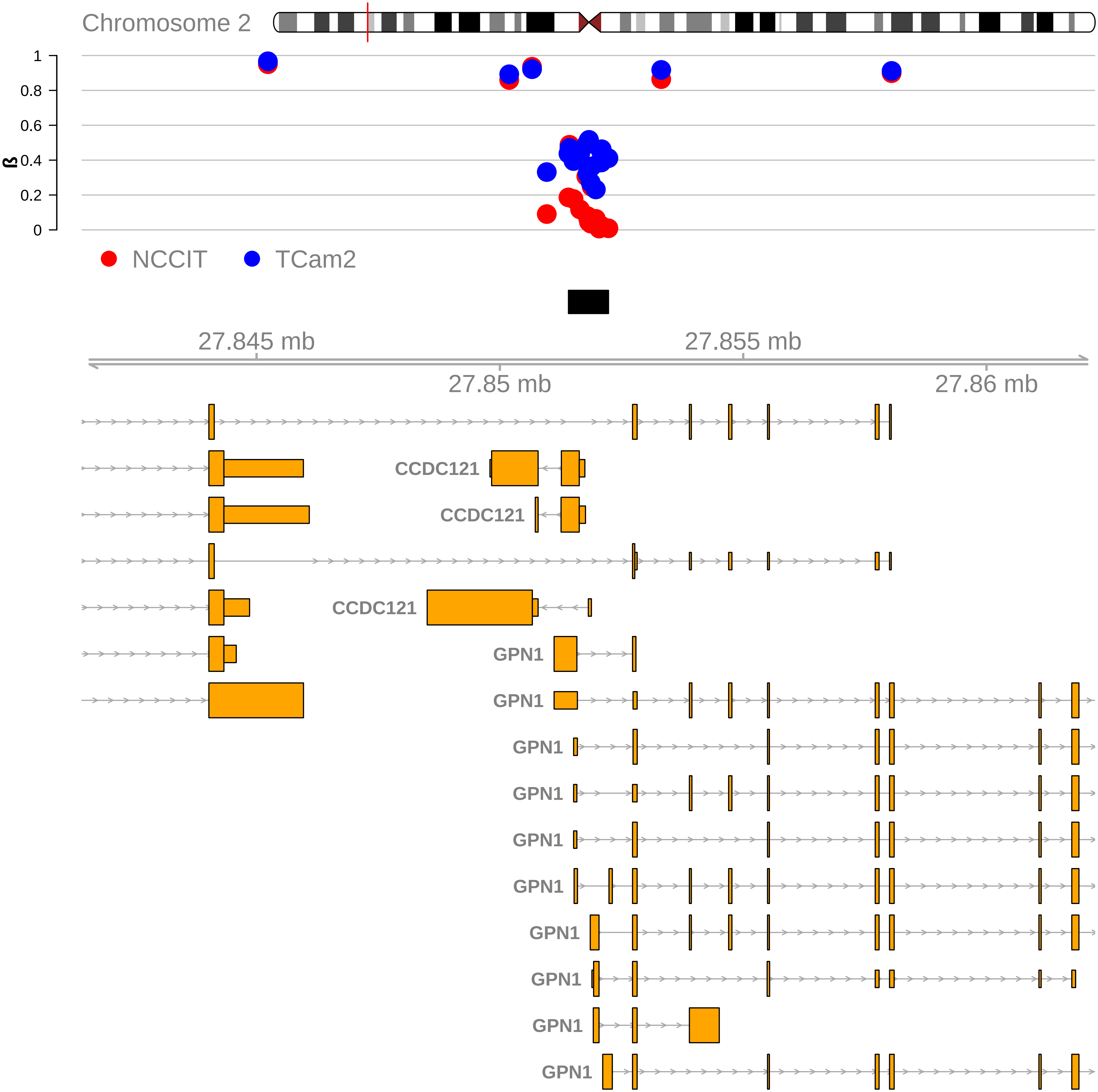

Chromosome 2
ß
1
0.8
0.6
0.4
0.2
0
NCCIT
TCam2
27.845 mb
27.855 mb
27.85 mb
27.86 mb
CCDC121
CCDC121
CCDC121
GPN1
GPN1
GPN1
GPN1
GPN1
GPN1
GPN1
GPN1
GPN1
GPN1
